# Supplementary material for: Environment scan of generative AI infrastructure for clinical and translational science
Source: Npj Health Syst. 2025 Jan 25;2:4. doi: 10.1038/s44401-024-00009-w (PMC11762411; doi:10.1038/s44401-024-00009-w)
Supplement: Supplementary file 1 — Supplementary materials [file 44401_2024_9_MOESM1_ESM.pdf]

Supplementary materials

A Questionnaire

Organization information

Organization name *:

Q1. Do you represent: (Select all that apply)

- a CTSA (academic institution)
- A hospital or healthcare system site
- Other (Please specify)

Stakeholder Identification and Roles

Q2. Which stakeholder groups are involved in your organization's decision-making and implementation of Generative AI? (Select all that apply)

- Hospital administration: Senior leaders (e.g., CEO, CMO, CIO, CMIO/CHIO, or CRIO)
- Hospital administration: Departmental leaders (e.g., medical directors, department chairs, or division chiefs)
- Hospital administration: Business unit leaders (e.g., nursing supervisors)
- Physicians
- Nurses
- Researchers
- IT staff (Data scientists, Data engineers, Health IT)
- Regulatory bodies (Legal, IRB)
- Policy groups (Local governance committees such as DEI committee and ethics committee)
- Patients
- Patient and community representatives
- Others (Please specify) ______

Q3. Who leads the decision-making process for implementing Generative AI applications in your organization? (Select all that apply)

- Hospital Administration with clinical expertise
- Cross-functional Committee (i.e., individuals with different backgrounds, roles, and expertise)
- Hospital Administration without clinical expertise
- IT Department
- Regulatory bodies
- Others (Please specify) ______

Decision-Making and Governance Structure

Q4. How are decisions regarding adopting Generative AI made in your healthcare system? (Select all that apply)

- Centralized (Top-down)
- Decentralized (Bottom-up; Individual departments independently decide to implement Generative AI based on their specific needs and experiences)
- Collaborative (Involving multiple departments)
- Others (Please specify) ______
- I don’t know

Q5. Are there any formal committees or task forces established to oversee the deployment and governance of Generative AI in your organization?

- Yes
  If yes, please describe the expertise and structure of the governance in your organization. ______
- No
- I don't know

Regulatory and Ethical Considerations

Q6. Which regulatory bodies are involved in overseeing the deployment of Generative AI in your organization? (Select all that apply)

- Federal agencies (e.g., FDA, HHS, DOC)
- State agencies
- Local Health Authorities
- Others

Q7. Do you have an ethicist or an ethics committee involved in the decision-making process for implementing Generative AI technologies in your organization?

- Yes, an ethicist is involved
- Yes, an ethics committee is involved
- No, neither an ethicist nor an ethics committee is involved
- I don't know

Q8. Please rank the following ethical considerations from most important (1) to least important (6) when decision-makers are deciding to implement Generative AI technologies:

- Bias and fairness of the generative AI algorithms
- Robustness of the generative AI algorithms
- Informed consent from patients
- Patient privacy and data security
- Transparency and explainability of AI decisions made by the algorithms
- Others (Please specify)

Stage of Adoption

Q9. What is the stage of generative AI adoption in your organization?

- Aware: Not actively considering Generative AI as a solution.
- Experimenting: Exploring the potential of Generative AI, building skills and expertise to improve communication, and identifying areas where Generative AI is adding value to the business
- Optimizing: Improving the AI literacy of your entire workforce, not just certain individuals or teams. A first solution is now running in production, as a patient-facing or mission-critical system.
- Standardizing: Ensuring standardized usage of gen AI across the business. Multiple solutions are now running in production.
- Transforming: maximizing the benefits in productivity, business communication, customer satisfaction, and the bottom line.

Q10. How well do Generative AI applications integrate with your existing systems and workflows? For CTSA, it could be OHDSI, ACT, etc. networks. For a healthcare system, it could be clinical workflow (e.g., EPIC).

- Very poorly

- Poorly
- Neutral
- Well
- Very well

Q11. How familiar are members of the workforce with the use of LLMs in your organization?

- Not at all familiar
- Sightly familiar
- Somewhat familiar
- Moderately familiar
- Extremely familiar

Q12. Has the workforce received any specific training related to LLMs in your organization?

- No, and not considered
- No, but considered
- Yes

Q13. How desirable is it for the workforce to receive further LLM training?

- Very undesirable
- Undesirable
- Neutral
- Desirable
- Very desirable

Q14. Is your institution working with a vendor to implement solutions?

- No, and not considered
- No, but considered
- Yes. Please indicate how many. ______

<u>Budget Trends</u>

Q15. Have funds been allocated for generative AI projects

- No, and not considered
- No, but considered
- Yes, systematically
- Yes, ad-hoc

Q16. Compared to 2021, the budget allocated to generative AI projects in your organization has:

- Increased by more than 300%
- Increased by 100-300%
- Increased by 50-100%
- Increased by 10-50%
- Remained roughly the same (±10%)
- Unknown

<u>Current LLM usage</u>

Q17. Which of the following types of LLM are you currently using? (Select all that apply)

- Untuned open LLMs (e.g., Llama, Falcon, and Mistral)

- Fine-tuned open LLMs (e.g., Llama, Falcon, and Mistral)
- Untuned proprietary LLMs (e.g., ChatGPT)
- Fine-tuned proprietary LLMs (e.g., ChatGPT)
- Combination of both open and proprietary LLMs
- Uncertain
- We are not using an LLM

Q18. What AI deployment options does your organization currently use?

- Cloud computing: Public cloud
- Cloud computing: Private cloud
- Cloud computing: Hybrid cloud
- On premises (self-hosting)
- Hybrid
- Others (Please specify) ______

Q19. You indicated that your organization is using open LLMs. What factors influenced your decision to develop internally? (Select all that apply)

- Technical architecture and deployment (e.g., LLM type, deployment options, infrastructure needs, data handling, compatibility)
- Data lifecycle management (e.g., data preprocessing, customer data use, data sharing, privacy, dedication features, sensitive information)
- Customization and integration features (e.g., proprietary datasets, third-party integrations)
- Security
- Scalability and performance
- LLM output compliance (e.g., bias, output filtering, diversity)
- Legal compliance (e.g., IP considerations, ownership)
- Regulatory compliance (e.g., consent, controls)
- Monitoring and reporting (e.g., transparency, accuracy, use feedback)
- Financial and operational considerations (e.g., costs, support, opt-out features)
- AI workforce development
- Clinician and/or patient buy-in
- Others (Please specify) ______

Q20. You indicated that your organization is using proprietary LLMs. What factors influenced your decision to go with commercial solutions? (Select all that apply)

- Technical architecture and deployment (e.g., LLM type, deployment options, infrastructure needs, data handling, compatibility)
- Data lifecycle management (e.g., data preprocessing, customer data use, data sharing, privacy, dedication features, sensitive information)
- Customization and integration features (e.g., proprietary datasets, third-party integrations)
- Security
- Scalability and performance
- LLM output compliance (e.g., bias, output filtering, diversity)
- Legal compliance (e.g., IP considerations, ownership)
- Regulatory compliance (e.g., consent, controls)
- Monitoring and reporting (e.g., transparency, accuracy, use feedback)
- Financial and operational considerations (e.g., costs, support, opt-out features)
- AI workforce development

- Clinician and/or patient buy-in
- Others (Please specify) ______

Q21. Which of the following use cases are you currently using LLMs for? (Select all that apply)

- Data abstraction
- Answering patient questions
- Biomedical research
- Clinical coding or chart audit
- De-identification
- Drug development
- Information extraction
- Machine translation
- Medical chatbot
- Medical image analysis
- Medical text summarization
- Natural language query interface
- Scheduling
- Synthetic data generation
- Transcribing medical encounters
- Others (Please specify) ______

Projected impact

Q22. On a scale of 1 to 5, please rate how much you think LLMs will impact each use case over the next 2-3 years. 1 means very negative, and 5 means very positive.

	1	2	3	4	5
Data abstraction					
Answering Patient Questions					
Biomedical Research					
Clinical Coding / Chart Audit					
De-identification					
Drug Development					
Information Extraction / Data					
Machine Translation					
Medical Chatbot					
Medical Image Analysis					
Medical Text Summarization					
Natural Language Query Interface					
Scheduling					
Synthetic Data Generation					
Transcribing Medical Encounters					

Q23. What improvements, if any, have you observed since implementing Generative AI solutions in your healthcare system? (Select all that apply)

- Better patient engagement
- Cost savings
- Enhanced diagnostic accuracy
- Faster decision-making processes

- Improved patient outcomes
- Increased operational efficiency
- Unknown
- Others (Please specify) ______

Q24. What drawbacks or negative impacts, if any, have you observed since implementing Generative AI solutions? (Select all that apply)

- Concerns about data security
- High maintenance costs
- Increased workload for staff
- Issues with AI bias
- Over-reliance on AI recommendations
- Lack of clinician trust
- Lack of patient trust
- It does not work well
- Unknown
- Others (Please specify) ______

LLM evaluation (if implementing LLM solutions)

Q25. On a scale from 1 to 5, please rate the importance of each of the following criteria when evaluating LLMs. 1 means "Not at all Important," and 5 means "Extremely Important."

	1	2	3	4	5
Accuracy					
Cost					
Explainability & Transparency					
Healthcare-Specific Models					
Legal & Reputational Risk					
Reproducible & Consistent Answers					
Security & Privacy Risk					

Q26. On a scale of 1 to 5, please rate how significant the following potential limitations or roadblocks are to your roadmap for current generative AI technology, with 1 being not important and 5 being very important.

	1	2	3	4	5
Compliance issue					
Falls Short on Bias & Fairness Requirements					
Lacks Accuracy					
Not Built for Healthcare & Life Science					
Not Tunable for Private Data or Use Cases					
Poses Legal, Security, or Reputational Risks					
Too Expensive					

Enhancement strategies

Q27. Which steps do you take to test and improve your LLM models? (Select all that apply)

- Adversarial testing
- De-biasing tools and techniques
- Guardrails
- Human in the loop

- Interpretability tools and techniques
- Quantization and/or Pruning
- Red Teaming
- Reinforcement Learning from Human Feedback (RLHF)
- Supervised fine-tuning
- Others (Please specify) ______
- None of the above

Q28. What type(s) of evaluations have your deployed LLM solutions undergone? (Select all that apply)

- Bias
- Brand Voice
- Explainability (citing sources)
- Fairness
- Freshness (data updates)
- Hallucinations / Disinformation
- Ideological Leaning
- Private Data Leakage
- Prompt Injection
- Robustness
- Sycophancy
- Toxicity
- Others (Please specify) ______
- None of the above

Q29. What challenges, if any, have you faced in integrating Generative AI with existing systems? (Select all that apply)

- Technical architecture and deployment (e.g., LLM type, deployment options, infrastructure needs, data handling, compatibility)
- Data lifecycle management (e.g., data preprocessing, customer data use, data sharing, privacy, dedication features, sensitive information)
- Customization and integration features (e.g., proprietary datasets, third-party integrations)
- Security
- Scalability and performance
- LLM output compliance (e.g., bias, output filtering, diversity)
- Legal and regulatory compliance (e.g., IP considerations, ownership, controls)
- Monitoring and reporting (e.g., transparency, accuracy, use feedback)
- Financial and operational considerations (e.g., costs, support, opt-out features)
- AI workforce development
- Others (Please specify) ______

Q30. Is there anything else you would like to add that was not covered in this survey? Please provide any additional comments, insights, or information that you believe is relevant to understanding the AI infrastructure within your organization.

————————————--

END OF SURVEY

Supplementary Table 1: Results of post-hoc McNemar tests with Bonferroni correction for stakeholder groups involved in the organization's decision-making and implementation of GenAI.

	Senior leaders	Departmental leaders	Business unit leaders	Clinicians	Nurses	Researchers	IT staff	Regulatory bodies	Policy groups	Patients	Patient representatives
p-value											
Departmental leaders	0.0117	-	-	-	-	-	-	-	-	-	-
Business unit leaders	<0.0001	0.0005	-	-	-	-	-	-	-	-	-
Clinicians	0.0018	0.5811	0.0127	-	-	-	-	-	-	-	-
Nurses	<0.0001	0.0001	0.7266	0.0010	-	-	-	-	-	-	-
Researchers	0.1797	0.3438	<0.0001	0.0391	<0.0001	-	-	-	-	-	-
IT staff	0.2500	0.1094	<0.0001	0.0117	<0.0001	0.7539	-	-	-	-	-
Regulatory bodies	0.0010	0.7744	0.0042	1.0000	0.0013	0.1460	0.0215	-	-	-	-
Policy groups	<0.0001	0.0923	0.0654	0.3877	0.0225	0.0034	0.0002	0.2266	-	-	-
Patients	<0.0001	<0.0001	0.0391	<0.0001	0.0625	<0.0001	<0.0001	<0.0001	0.0001	-	-
Patient representatives	<0.0001	<0.0001	0.1797	0.0009	0.4531	<0.0001	<0.0001	<0.0001	0.0018	0.6250	
Others	<0.0001	<0.0001	0.0654	0.0001	0.2266	<0.0001	<0.0001	<0.0001	0.0013	1.0000	0.6875
Corrrected p-value											
Departmental leaders	0.7734	-	-	-	-	-	-	-	-	-	-
Business unit leaders	<0.0001	0.0342	-	-	-	-	-	-	-	-	-
Clinicians	0.1208	1.0000	0.8399	-	-	-	-	-	-	-	-
Nurses	<0.0001	0.0096	1.0000	0.0645	-	-	-	-	-	-	-
Researchers	1.0000	1.0000	0.0005	1.0000	0.0001	-	-	-	-	-	-
IT staff	1.0000	1.0000	0.0001	0.7734	<0.0001	1.0000	-	-	-	-	-
Regulatory bodies	0.0645	1.0000	0.2759	1.0000	0.0866	1.0000	1.0000	-	-	-	-
Policy groups	0.0020	1.0000	1.0000	1.0000	1.0000	0.2256	0.0161	1.0000	-	-	-
Patients	<0.0001	<0.0001	1.0000	0.0026	1.0000	<0.0001	<0.0001	0.0003	0.0081	-	-
Patient representatives	<0.0001	0.0003	1.0000	0.0565	1.0000	<0.0001	<0.0001	0.0010	0.1208	1.0000	-
Others	<0.0001	<0.0001	1.0000	0.0080	1.0000	<0.0001	<0.0001	0.0003	0.0866	1.0000	1.0000

Supplementary Table 2: Results of post-hoc McNemar tests with Bonferroni correction for leaders of the decision-making process for implementing GenAI applications in the organization.

	Clinical Leadership	Cross-functional Committee	Hospital Administration	IT Department	Regulatory bodies
p-value					
Cross-functional Committee	0.0127	-	-	-	-
Hospital Administration	0.7266	0.0023	-	-	-
IT Department	1.0000	0.0075	1.0000	-	-
Regulatory bodies	0.0018	<0.0001	0.0129	0.0074	-
Others	0.0075	<0.0001	0.0414	0.0192	1.0000
Corrrected p-value					
Cross-functional Committee	0.1909	-	-	-	-
Hospital Administration	1.0000	0.0352	-	-	-
IT Department	1.0000	0.1131	1.0000	-	-
Regulatory bodies	0.0275	<0.0001	0.1941	0.1108	-
Others	0.1131	0.0005	0.6208	0.2882	1.0000

Supplementary Table 3: Excerpts of governance and leadership structures in GenAI deployment across CTSA institutions.

**Free Text Answers**
Clinical, IT, machine learning, for the most part
We have multiple governance committees. We have one focused on the university and non-clinical applications. We have a separate joint governance structure that includes representation from the medical school and health system leadership. Both sets of governance includes informatics leadership and those who are conducting research in Generative AI and would be considered subject matter experts. It also has IT representation from the perspective of supporting generative AI infrastructure, access, and policies. Our governance structure also incorporates clinical and/or academic leadership.
Health Data Oversight Committee - oversees Data Acesss Committee, Health Analytics Committee, Data Sharing Committee, Advanced Computing Committee. Each of these subcommitees have representation from clinical/research faculty and IT. HDOC itself includes Dean, VD and senior IT/ roles, and report up to the Vice Chancellor for Human Health Sciences.
Committee chaired by the Provost with representatives from Clinical Informatics, Chair of Biomedical informatics, Clinical and Research leadership
Healthcare system and School of Medicine have an integrated IT governance system that makes decisions about new technology to be adopted.
We have a new AI governance group, generative AI is loosely in the scope of this group.
Senior leadership establishes policy and has created an generative AI committee and has partnered with Microsoft to allow OpenAI models to be run at [institution]. However, they disallow identified data to be submitted to Open AI LLM endpoints limiting clinical application. The generative AI committee has nobody with deep AI experience (Computer Science PhD in relevant field or any Computer Scientists at all as far as I know). Many Departments are experimenting with generative AI, writing papers, proposing research, including trying some biomedical and clinical applications. All implementation and experiments are bottom up, but policy and the Microsoft partnership is top-down.
The university has a governance committee of Generative AI that CTSA participates.
Clinical AI strategic planning group (clinical, admin, data science, IT) sets direction, Responsible AI Oversight Group provides governance
We have operational and regulatory leaders from IT, quality analytics, CISO, CMIO, associate dean for informatics, associate dean for compliance
It is within scope for our standing Clinical Artificial Intelligence Committee which evaluates AI-based algorithms and interventions
IT and CRIO work collaboratively on the governance of Gen AI.
AI Governance committee that guides strategy and completes intake for requests.
Work in progress - vetting of tools, regulatory review processes
Committee on general AI, including generative
Institution has established a number of committees governing implementation of AI. These include the AI Committee Supporting Teaching, Learning, & Discovery; the AI Risks, Ethics and Policy Committee; and the Institution HS Artificial Intelligence and Machine Learning Governance (AIGOV) Committee.
Knowledge rooted in IT, computer science, informatics, clinicians
CMIO, clinician champions, regulatory representatives, IT
We have an Enterprise AI Translation Advisory Board led by seasoned clinical informaticians, health IT professionals, data scientists, AI researchers, and legal experts that represent organization executives, IT operation & governance, technology innovation & translation, health care delivery, as well as ethics and compliance.
See prior responses
We have University-wide multi-discipinary work group and a Hosptial-based CMIO lead workgroup
The committee continues to evolve but includes clinical, EHR, and IT leadership as well as research leadership where necessary.
We have a few tri-institutional governance committees that oversee GenAI implementation. We have a clinical group that reviews utility, another committee that evaluates models and another that reviews health equity.

Continued on next page

Supplementary Table 3 – continued from previous page.

**Free Text Answers**
There is strategic and operational governance of genAI deployed in the Health system. Operational governance includes clinicians and researchers with genAI expertise. Outside of the health system, operational governance is partially in the IRB and in IT Governance committees that do not have sufficient expertise. Strategic governance of non-Health system genAI is at the Chancellor's level and not formally chartered.
Institution consists of two separate legal entities, the University and its Health System. These two institutions have separate IT and governance structures. To be able to conduct biomedical research, especially using clinical data, we have a research data governance structure that negotiates the rules from the two systems. This committee is also working on data and infrastructure sharing with our partner institutions (We are a multi-institutional CTSA).
Governance Committee on Data, AI and Analytics
The Generative AI Healthcare Workgroup is a broad team with representation from across the hospital and including some clinician researchers and clinical informaticists. The workgroup is led by the CMIO.
Clinical informatics leaders with representatives from departments/IT

Supplementary Table 4: Results of post-hoc McNemar tests with Bonferroni correction for current use of LLMs.

	We are not using an LLM	Combination of both	Open LLMs only
P-value			
Combination of both	<0.0001	-	-
Open LLMs only	0.3750	0.0005	-
Proprietary LLMs only	0.0215	0.0294	0.2668
Corrrected p-value			
Combination of both	<0.0001	-	-
Open LLMs only	1.0000	0.0032	-
Proprietary LLMs only	0.1289	0.1767	1.0000

Supplementary Table 5: Results of post-hoc McNemar tests with Bonferroni correction for factors influenced the decision to develop internally when the organization uses open LLMs

	Technical architecture and deployment	Data lifecycle management	Customization and integration features	Security	Scalability and performance	LLM output compliance	Legal compliance	Regulatory compliance	Monitoring and reporting	Financial and operational considerations	AI workforce development	Clinician and/or patient buy-in
p-value												
Data lifecycle management	0.1797	-	-	-	-	-	-	-	-	-	-	-
Customization and integration features	0.3438	1.0000	-	-	-	-	-	-	-	-	-	-
Security	0.3018	1.0000	1.0000	-	-	-	-	-	-	-	-	-
Scalability and performance	0.0023	0.0963	0.0490	0.0963	-	-	-	-	-	-	-	-
LLM output compliance	0.0127	0.1460	0.0923	0.1460	0.7905	-	-	-	-	-	-	-
Legal compliance	0.0574	0.4531	0.2891	0.4531	0.3018	0.5078	-	-	-	-	-	-
Regulatory compliance	0.1460	1.0000	0.7744	1.0000	0.1435	0.2266	0.7266	-	-	-	-	-
Monitoring and reporting	0.0213	0.2266	0.1094	0.2266	0.5811	1.0000	0.6875	0.2891	-	-	-	-
Financial and operational considerations	0.2266	1.0000	1.0000	1.0000	0.0574	0.1796	0.5811	1.0000	0.3018	-	-	-
AI workforce development	0.0063	0.1797	0.1460	0.3018	0.6072	1.0000	0.7744	0.3438	1.0000	0.3018	-	-
Clinician and/or patient buy-in	<0.0001	0.0010	0.0005	0.0074	0.5488	0.1250	0.0215	0.0063	0.0313	0.0034	0.0313	-
Others	0.0013	0.0784	0.0309	0.0931	1.0000	0.6291	0.2632	0.1153	0.4807	0.0352	0.4807	0.7905
Corrrected p-value												
Data lifecycle management	1.0000	-	-	-	-	-	-	-	-	-	-	-
Customization and integration features	1.0000	1.0000	-	-	-	-	-	-	-	-	-	-
Security	1.0000	1.0000	1.0000	-	-	-	-	-	-	-	-	-
Scalability and performance	0.1833	1.0000	1.0000	1.0000	-	-	-	-	-	-	-	-
LLM output compliance	0.9926	1.0000	1.0000	1.0000	1.0000	-	-	-	-	-	-	-
Legal compliance	1.0000	1.0000	1.0000	1.0000	1.0000	1.0000	-	-	-	-	-	-
Regulatory compliance	1.0000	1.0000	1.0000	1.0000	1.0000	1.0000	1.0000	-	-	-	-	-
Monitoring and reporting	1.0000	1.0000	1.0000	1.0000	1.0000	1.0000	1.0000	1.0000	-	-	-	-
Financial and operational considerations	1.0000	1.0000	1.0000	1.0000	1.0000	1.0000	1.0000	1.0000	1.0000	-	-	-
AI workforce development	0.4951	1.0000	1.0000	1.0000	1.0000	1.0000	1.0000	1.0000	1.0000	1.0000	-	-
Clinician and/or patient buy-in	0.0024	0.0762	0.0381	0.5760	1.0000	1.0000	1.0000	0.4951	1.0000	0.2666	1.0000	-
Others	0.1024	1.0000	1.0000	1.0000	1.0000	1.0000	1.0000	1.0000	1.0000	1.0000	1.0000	1.0000

Supplementary Table 6: Results of post-hoc McNemar tests with Bonferroni correction for approaches used in GenAI deployment.

	Public cloud	Private cloud	Hybrid cloud	On premises	Hybrid
P-value					
Private cloud	0.0001	-	-	-	-
Hybrid cloud	1.0000	0.0026	-	-	-
On premises	0.0004	1.0000	0.0015	-	-
Hybrid	0.7539	0.0043	1.0000	0.0026	-
Others	0.5488	$<$0.0001	0.3438	$<$0.0001	0.2266
Corrrected p-value					
Private cloud	0.0022	-	-	-	-
Hybrid cloud	1.0000	0.0390	-	-	-
On premises	0.0060	1.0000	0.0223	-	-
Hybrid	1.0000	0.0652	1.0000	0.0387	-
Others	1.0000	0.0010	1.0000	0.0003	1.0000

Supplementary Table 7: Results of post-hoc McNemar tests with Bonferroni correction for factors that influenced the decision to go with proprietary LLMs.

	Technical architecture and deployment	Data lifecycle management	Customization and integration features	Security	Scalability and performance	LLM output compliance	Legal compliance	Regulatory compliance	Monitoring and reporting	Financial and operational considerations	AI workforce development	Clinician and/or patient buy-in
p-value												
Data lifecycle management	0.0018	-	-	-	-	-	-	-	-	-	-	-
Customization and integration features	0.0117	0.5811	-	-	-	-	-	-	-	-	-	-
Security	0.1796	0.0703	0.5811	-	-	-	-	-	-	-	-	-
Scalability and performance	0.7905	0.0129	0.1435	0.4240	-	-	-	-	-	-	-	-
LLM output compliance	0.0004	0.3438	0.0654	0.0213	0.0005	-	-	-	-	-	-	-
Legal compliance	0.0023	1.0000	0.4545	0.0654	0.0192	0.5078	-	-	-	-	-	-
Regulatory compliance	0.0490	0.5811	1.0000	0.6072	0.1435	0.0654	0.2891	-	-	-	-	-
Monitoring and reporting	0.0001	0.3438	0.1185	0.0213	0.0005	1.0000	0.5488	0.0654	-	-	-	-
Financial and operational considerations	0.2101	0.1796	0.6072	1.0000	0.4240	0.0063	0.1185	0.5488	0.0309	-	-	-
AI workforce development	0.0001	0.3438	0.0654	0.0129	0.0001	1.0000	0.5488	0.0654	1.0000	0.0020	-	-
Clinician and/or patient buy-in	0.0005	0.7539	0.3018	0.0386	0.0075	0.7539	1.0000	0.3018	0.7539	0.0574	0.7539	-
Others	0.0002	0.2668	0.0574	0.0192	0.0007	1.0000	0.3877	0.0768	1.0000	0.0192	1.0000	0.5078
Corrrected p-value												
Data lifecycle management	0.1428	-	-	-	-	-	-	-	-	-	-	
Customization and integration features	0.9141	1.0000	-	-	-	-	-	-	-	-	-	
Security	1.0000	1.0000	1.0000	-	-	-	-	-	-	-	-	
Scalability and performance	1.0000	1.0000	1.0000	1.0000	-	-	-	-	-	-	-	
LLM output compliance	0.0314	1.0000	1.0000	1.0000	0.0405	-	-	-	-	-	-	
Legal compliance	0.1833	1.0000	1.0000	1.0000	1.0000	1.0000	-	-	-	-	-	
Regulatory compliance	1.0000	1.0000	1.0000	1.0000	1.0000	1.0000	1.0000	-	-	-	-	
Monitoring and reporting	0.0113	1.0000	1.0000	1.0000	0.0405	1.0000	1.0000	1.0000	-	-	-	
Financial and operational considerations	1.0000	1.0000	1.0000	1.0000	1.0000	0.4951	1.0000	1.0000	1.0000	-	-	
AI workforce development	0.0113	1.0000	1.0000	1.0000	0.0095	1.0000	1.0000	1.0000	1.0000	0.1523	-	
Clinician and/or patient buy-in	0.0405	1.0000	1.0000	1.0000	0.5880	1.0000	1.0000	1.0000	1.0000	1.0000	1.0000	
Others	0.0173	1.0000	1.0000	1.0000	0.0568	1.0000	1.0000	1.0000	1.0000	1.0000	1.0000	1.0000

Supplementary Table 8: Results of Co-Occurrence Analysis for factors influenced the decision to develop internally when the organization uses open LLMs.

-	Data abstraction	Answering patient questions	Biomedical research	Clinical coding or chart audit	De-identification	Drug development	Information extraction	Machine translation	Medical chatbot	Medical image analysis	Medical text summarization	Natural language query interface	Scheduling	Synthetic data generation	Transcribing medical encounters
Answering patient questions	10	-	-	-	-	-	-	-	-	-	-	-	-	-	-
Biomedical research	17	7	-	-	-	-	-	-	-	-	-	-	-	-	-
Clinical coding or chart audit	11	5	12	-	-	-	-	-	-	-	-	-	-	-	-
De-identification	8	3	10	4	-	-	-	-	-	-	-	-	-	-	-
Drug development	3	0	4	3	1	-	-	-	-	-	-	-	-	-	-
Information extraction	16	9	18	10	9	2	-	-	-	-	-	-	-	-	-
Machine translation	3	1	3	2	1	0	3	-	-	-	-	-	-	-	-
Medical chatbot	10	10	9	7	3	1	10	2	-	-	-	-	-	-	-
Medical image analysis	8	5	11	7	5	4	10	3	6	-	-	-	-	-	-
Medical text summarization	18	11	18	10	8	3	15	4	10	10	-	-	-	-	-
Natural language query interface	12	7	13	6	6	2	11	4	9	8	12	-	-	-	-
Scheduling	1	1	0	0	0	0	1	0	1	0	1	0	-	-	-
Synthetic data generation	7	4	8	4	5	1	9	0	5	7	7	7	0	-	-
Transcribing medical encounters	11	9	11	7	6	2	11	2	6	7	13	7	1	4	-
Other	2	1	2	1	1	0	2	2	1	0	0	0	0	0	2

Supplementary Table 9: Results of post-hoc McNemar tests with Bonferroni correction for LLM use cases.

	Data abstraction	Answering patient questions	Biomedical research	Clinical coding or chart audit	De-identification	Drug development	Information extraction	Machine translation	Medical chatbot	Medical image analysis	Medical text summarization	Natural language query interface	Scheduling	Synthetic data generation	Transcribing medical encounters
p-value															
Answering patient questions	0.0490	-	-	-	-	-	-	-	-	-	-	-	-	-	-
Biomedical research	1.0000	0.0639	-	-	-	-	-	-	-	-	-	-	-	-	-
Clinical coding or chart audit	0.0129	1.0000	0.0034	-	-	-	-	-	-	-	-	-	-	-	-
De-identification	0.0023	0.4807	0.0001	0.6072	-	-	-	-	-	-	-	-	-	-	-
Drug development	<0.0001	0.0309	<0.0001	0.0117	0.1460	-	-	-	-	-	-	-	-	-	-
Information extraction	0.7744	0.1435	0.5078	0.0574	0.0034	0.0002	-	-	-	-	-	-	-	-	-
Machine translation	<0.0001	0.0213	<0.0001	0.0225	0.1460	1.0000	<0.0001	-	-	-	-	-	-	-	-
Medical chatbot	0.0213	1.0000	0.0192	1.0000	0.6291	0.0352	0.0574	0.0225	-	-	-	-	-	-	-
Medical image analysis	0.0414	1.0000	0.0074	1.0000	0.5811	0.0039	0.0574	0.0117	1.0000	-	-	-	-	-	-
Medical text summarization	1.0000	0.0213	1.0000	0.0127	0.0013	<0.0001	0.6072	<0.0001	0.0127	0.0127	-	-	-	-	-
Natural language query interface	0.0574	1.0000	0.0225	0.8036	0.2668	0.0074	0.1796	0.0010	0.7539	0.7744	0.0352	-	-	-	-
Scheduling	<0.0001	0.0002	<0.0001	0.0018	0.0117	0.3750	<0.0001	0.3750	0.0005	0.0018	<0.0001	0.0005	-	-	-
Synthetic data generation	0.0013	0.3018	0.0003	0.4240	1.0000	0.2266	0.0005	0.1797	0.3877	0.2891	0.0007	0.1094	0.0215	-	-
Transcribing medical encounters	0.2379	0.5811	0.1671	0.4545	0.1185	0.0023	0.4545	0.0023	0.4807	0.4545	0.1185	0.8145	<0.0001	0.0963	-
Other	0.0003	0.0490	0.0002	0.0768	0.2668	1.0000	0.0009	1.0000	0.0768	0.0963	0.0005	0.0309	0.2188	0.4240	0.00750
Corrrected p-value															
Answering patient questions	1.0000	-	-	-	-	-	-	-	-	-	-	-	-	-	-
Biomedical research	1.0000	1.0000	-	-	-	-	-	-	-	-	-	-	-	-	-
Clinical coding or chart audit	1.0000	1.0000	0.4102	-	-	-	-	-	-	-	-	-	-	-	-
De-identification	0.2820	1.0000	0.0146	1.0000	-	-	-	-	-	-	-	-	-	-	-
Drug development	0.0025	1.0000	0.0002	1.0000	1.0000	-	-	-	-	-	-	-	-	-	-
Information extraction	1.0000	1.0000	1.0000	1.0000	0.4102	0.0266	-	-	-	-	-	-	-	-	-
Machine translation	0.0025	1.0000	0.0013	1.0000	1.0000	1.0000	0.0092	-	-	-	-	-	-	-	-
Medical chatbot	1.0000	1.0000	1.0000	1.0000	1.0000	1.0000	1.0000	1.0000	-	-	-	-	-	-	-
Medical image analysis	1.0000	1.0000	0.8862	1.0000	1.0000	0.4688	1.0000	1.0000	1.0000	-	-	-	-	-	-
Medical text summarization	1.0000	1.0000	1.0000	1.0000	0.1575	0.0013	1.0000	0.0002	1.0000	1.0000	-	-	-	-	-
Natural language query interface	1.0000	1.0000	1.0000	1.0000	1.0000	0.8862	1.0000	0.1172	1.0000	1.0000	1.0000	-	-	-	-
Scheduling	<0.0001	0.0293	0.0002	0.2197	1.0000	1.0000	0.0002	1.0000	0.0586	0.2197	<0.0001	0.0623	-	-	-
Synthetic data generation	0.1575	1.0000	0.0330	1.0000	1.0000	1.0000	0.0586	1.0000	1.0000	1.0000	0.0874	1.0000	1.0000	-	-
Transcribing medical encounters	1.0000	1.0000	1.0000	1.0000	1.0000	0.2820	1.0000	0.2820	1.0000	1.0000	1.0000	1.0000	0.0037	1.0000	-
Other	0.0333	1.0000	0.0188	1.0000	1.0000	1.0000	0.1027	1.0000	1.0000	1.0000	0.0655	1.0000	1.0000	1.0000	0.9045

Supplementary Table 10: Mean ratings of important criteria when evaluating LLMs.

**Use Case**	**Mean Rating**
Use Case	4.5278
Accuracy	4.4722
Reproducible & Consistent Answers	4.4444
Security & Privacy Risk	4.1944
Legal & Reputational Risk	4.0556
Cost	3.9167
Healthcare-Specific Models	3.8611
Explainability & Transparency	3.6111

Supplementary Table 11: Mean ratings of significant limitation for current GenAI technology.

Use Case	Mean Rating
Compliance issue	4.2222
Lacks Accuracy	4.1389
Poses Legal, Security, or Reputational Risks	4.0278
Too Expensive	3.9444
Falls Short on Bias & Fairness Requirements	3.9167
Not Built for Healthcare & Life Science	3.6389
Not Tunable for Private Data or Use Cases	3.5833

Supplementary Table 12: Mean ratings of how much LLMs will impact each use case over the next 2-3 years.

**Use Case**	**Mean Rating**
Natural Language Query Interface	4.5556
Information Extraction / Data	4.5000
Medical Text Summarization	4.4722
Transcribing Medical Encounters	4.3333
Data Abstraction	4.2778
Medical Image Analysis	4.1944
Biomedical Research	4.1111
Machine Translation	4.0278
Clinical Coding / Chart Audit	3.9167
Medical Chatbot	3.9167
Answering Patient Questions	3.7778
De-identification	3.6944
Synthetic Data Generation	3.5556
Scheduling	3.5278
Drug Development	3.3889
Others	3.2222

Supplementary Table 13: Results of post-hoc McNemar tests with Bonferroni correction for improvements observed since implementing GenAI solutions in the healthcare system.

	Better patient engagement	Cost savings	Enhanced diagnostic accuracy	Faster decision-making processes	Improved patient outcomes	Increased operational efficiency	N/A - Have not started
p-value							
Cost savings	0.1250	-	-	-	-	-	-
Enhanced diagnostic accuracy	0.3750	0.6250	-	-	-	-	-
Faster decision-making processes	0.3750	0.0039	0.0414	-	-	-	-
Improved patient outcomes	0.0001	0.1338	0.3438	1.0000	-	-	-
Increased operational efficiency	0.0023	0.7905	0.0010	0.1892	N/A	-	-
N/A - Have not started	0.0391	1.0000	0.0414	0.2632	N/A	N/A	-
Others	0.6875	0.0034	0.3877	N/A	N/A	N/A	N/A
Corrrected p-value							
Cost savings	1.0000	-	-	-	-	-	-
Enhanced diagnostic accuracy	1.0000	1.0000	-	-	-	-	-
Faster decision-making processes	1.0000	0.0820	0.8692	-	-	-	-
Improved patient outcomes	0.0026	1.0000	1.0000	1.0000	-	-	-
Increased operational efficiency	0.0493	1.0000	0.0205	1.0000	N/A	-	-
N/A - Have not started	0.8203	1.0000	0.8692	1.0000	N/A	N/A	-
Others	1.0000	0.0718	1.0000	N/A	N/A	N/A	N/A

Supplementary Table 14: Results of post-hoc McNemar tests with Bonferroni correction for steps to test and improve LLM models. RLHF - Reinforcement Learning from Human Feedback.

	Adversarial testing	De-biasing tools and techniques	Guardrails	Human in the loop	Interpretability tools and techniques	Quantization and/or Pruning	Red Teaming	RLHF	Supervised fine-tuning	Others
p-value										
De-biasing tools and techniques	0.0074	-	-	-	-	-	-	-	-	-
Guardrails	0.0386	0.5811	-	-	-	-	-	-	-	-
Human in the loop	<0.0001	0.0034	0.0001	-	-	-	-	-	-	-
Interpretability tools and techniques	0.0127	1.0000	0.6291	0.0034	-	-	-	-	-	-
Quantization and/or Pruning	1.0000	0.0034	0.0386	<0.0001	0.0010	-	-	-	-	-
Red Teaming	0.6875	0.0010	0.0020	<0.0001	0.0010	0.6875	-	-	-	-
RLHF	0.0654	0.3438	1.0000	<0.0001	0.4240	0.0654	0.0225	-	-	-
Supervised fine-tuning	0.0005	1.0000	0.4545	0.0020	1.0000	0.0018	0.0005	0.2266	-	-
Others	0.7539	0.0044	0.0309	<0.0001	0.0044	0.7266	1.0000	0.0352	0.0026	-
None of the above	0.1094	0.0002	0.0013	<0.0001	0.0002	0.1094	0.2891	0.0023	0.0001	0.2891
Corrrected p-value										
De-biasing tools and techniques	0.4062	-	-	-	-	-	-	-	-	-
Guardrails	1.0000	1.0000	-	-	-	-	-	-	-	-
Human in the loop	<0.0001	0.1880	0.0067	-	-	-	-	-	-	-
Interpretability tools and techniques	0.6999	1.0000	1.0000	0.1880	-	-	-	-	-	-
Quantization and/or Pruning	1.0000	0.1880	1.0000	<0.0001	0.0537	-	-	-	-	-
Red Teaming	1.0000	0.0537	0.1074	<0.0001	0.0537	1.0000	-	-	-	-
RLHF	1.0000	1.0000	1.0000	0.0034	1.0000	1.0000	1.0000	-	-	-
Supervised fine-tuning	0.0269	1.0000	1.0000	0.1074	1.0000	0.1007	0.0285	1.0000	-	-
Others	1.0000	0.2434	1.0000	0.0005	0.2434	1.0000	1.0000	1.0000	0.1417	-
None of the above	1.0000	0.0122	0.0722	<0.0001	0.0122	1.0000	1.0000	0.1292	0.0067	1.0000

Supplementary Table 15: Results of post-hoc McNemar tests with Bonferroni correction for type(s) of evaluations when deployed LLM solutions.

	Bias	Brand Voice	Explainability	Fairness	Freshness	Hallucinations / Disinformation	Ideological Leaning	Pruvate Data Leakage	Prompt Injection	Robustness	Sycophancy	Toxicity	Others
p-value													
Brand Voice	0.7266	-	-	-	-	-	-	-	-	-	-	-	-
Explainability	0.0313	0.0313	-	-	-	-	-	-	-	-	-	-	-
Fairness	0.0034	0.0117	0.0625	-	-	-	-	-	-	-	-	-	-
Freshness	0.3438	0.3438	0.1094	0.2266	-	-	-	-	-	-	-	-	-
Hallucinations / Disinformation	0.0034	0.0117	0.2188	0.3438	1.0000	-	-	-	-	-	-	-	-
Ideological Leaning	0.0703	0.7744	1.0000	<0.0001	0.0034	1.0000	-	-	-	-	-	-	-
Private Data Leakage	0.0010	0.0078	1.0000	0.0129	1.0000	0.0034	0.3438	-	-	-	-	-	-
Prompt Injection	1.0000	0.4545	<0.0001	<0.0001	0.1797	1.0000	0.5488	N/A	-	-	-	-	-
Robustness	0.0063	0.3593	1.0000	0.3438	0.3438	0.1797	1.0000	N/A	N/A	-	-	-	-
Sycophancy	0.2632	0.1797	0.1250	0.0001	0.1250	0.3438	N/A	N/A	N/A	N/A	-	-	-
Toxicity	0.1892	0.0020	1.0000	0.0525	0.1460	0.0063	N/A	N/A	N/A	N/A	N/A	-	-
Others	0.2188	0.1797	0.0010	0.0433	0.2891	0.2863	N/A	N/A	N/A	N/A	N/A	N/A	-
None of the above	0.0117	1.0000	1.0000	0.1250	1.0000	0.1892	N/A	N/A	N/A	N/A	N/A	N/A	N/A
Corrrected p-value													
Brand Voice	1.0000	-	-	-	-	-	-	-	-	-	-	-	-
Explainability	1.0000	1.0000	-	-	-	-	-	-	-	-	-	-	-
Fairness	0.2256	0.7734	1.0000	-	-	-	-	-	-	-	-	-	-
Freshness	1.0000	1.0000	1.0000	1.0000	-	-	-	-	-	-	-	-	-
Hallucinations / Disinformation	0.2256	0.7734	1.0000	1.0000	1.0000	-	-	-	-	-	-	-	-
Ideological Leaning	1.0000	1.0000	1.0000	0.0040	0.2256	1.0000	-	-	-	-	-	-	-
Private Data Leakage	0.0645	0.5156	1.0000	0.8540	1.0000	0.2256	1.0000	-	-	-	-	-	-
Prompt Injection	1.0000	1.0000	0.0040	0.0040	1.0000	1.0000	1.0000	N/A	-	-	-	-	-
Robustness	0.4189	1.0000	1.0000	1.0000	1.0000	1.0000	1.0000	N/A	N/A	-	-	-	-
Sycophancy	1.0000	1.0000	1.0000	0.0081	1.0000	1.0000	N/A	N/A	N/A	N/A	-	-	-
Toxicity	1.0000	0.1289	1.0000	1.0000	1.0000	0.4189	N/A	N/A	N/A	N/A	N/A	-	-
Others	1.0000	1.0000	0.0645	1.0000	1.0000	1.0000	N/A	N/A	N/A	N/A	N/A	N/A	-
None of the above	0.7734	1.0000	1.0000	1.0000	1.0000	1.0000	N/A	N/A	N/A	N/A	N/A	N/A	N/A

Supplementary Table 16: Results of post-hoc McNemar tests with Bonferroni correction for challenges faced in integrating GenAI with existing systems.

	Technical architecture and deployment	Data lifecycle management	Customization and integration features	Security	Scalability and performance	LLM output compliance	Legal and regulatory compliance	Monitoring and reporting	Financial and operational considerations	AI workforce development
p-value										
Data lifecycle management	0.1185	-	-	-	-	-	-	-	-	-
Customization and integration features	0.1796	1.0000	-	-	-	-	-	-	-	-
Security	0.1460	1.0000	1.0000	-	-	-	-	-	-	-
Scalability and performance	0.0005	0.3018	0.2101	0.2101	-	-	-	-	-	-
LLM output compliance	0.0005	0.3018	0.1796	0.1796	1.0000	-	-	-	-	-
Legal and regulatory compliance	0.0192	0.3438	0.2266	0.2668	1.0000	1.0000	-	-	-	-
Monitoring and reporting	0.0225	0.7744	0.5488	0.6072	0.5488	0.5488	0.7539	-	-	-
Financial and operational considerations	0.0117	0.7744	0.5488	0.5488	0.5488	0.6072	0.7539	1.0000	-	-
AI workforce development	0.0768	1.0000	0.7744	0.7905	0.3877	0.4545	0.5488	1.0000	1.0000	-
Others	<0.0001	0.0004	0.0002	0.0002	0.0127	0.0127	0.0075	0.0026	0.0026	0.0015
Corrrected p-value										
Data lifecycle management	1.0000	-	-	-	-	-	-	-	-	-
Customization and integration features	1.0000	1.0000	-	-	-	-	-	-	-	-
Security	1.0000	1.0000	1.0000	-	-	-	-	-	-	-
Scalability and performance	0.0269	1.0000	1.0000	1.0000	-	-	-	-	-	-
LLM output compliance	0.0269	1.0000	1.0000	1.0000	1.0000	-	-	-	-	-
Legal and regulatory compliance	1.0000	1.0000	1.0000	1.0000	1.0000	1.0000	-	-	-	-
Monitoring and reporting	1.0000	1.0000	1.0000	1.0000	1.0000	1.0000	1.0000	-	-	-
Financial and operational considerations	0.6445	1.0000	1.0000	1.0000	1.0000	1.0000	1.0000	1.0000	-	-
AI workforce development	1.0000	1.0000	1.0000	1.0000	1.0000	1.0000	1.0000	1.0000	1.0000	-
Others	0.0008	0.0221	0.0122	0.0122	0.6999	0.6999	0.4146	0.1417	0.1417	0.0819
